# Humanistic outcomes in treatment resistant depression: a secondary analysis of the STAR*D study

**DOI:** 10.1186/s12888-018-1920-7

**Published:** 2018-10-29

**Authors:** Allitia DiBernardo, Xiwu Lin, Qiaoyi Zhang, Jim Xiang, Lang Lu, Carol Jamieson, Carmela Benson, Kwan Lee, Robert Bodén, Lena Brandt, Philip Brenner, Johan Reutfors, Gang Li

**Affiliations:** 1 Janssen Research & Development, LLC, Titusville, NJ USA; 2 Janssen Scientific Affairs, LLC, Titusville, NJ USA; 3 Department of Neuroscience, Psychiatry, Uppsala University, Uppsala, Sweden; 4 Centre for Pharmacoepidemiology, Clinical Epidemiology, Department of Medicine Solna, Karolinska Institutet, Karolinska University Hospital, Stockholm, Sweden; 5 Real World Evidence, Statistics & Decision Sciences, Janssen R&D US, 920 US Highway 202 S, Raritan, NJ 08869 USA

**Keywords:** Health-related quality of life, Humanistic outcomes, Social functioning, Star*d, Treatment resistant depression

## Abstract

**Background:**

In the Sequenced Treatment Alternatives to Relieve Depression (STAR*D) study, a third of patients did not achieve remission or adequate response after two treatment trials, fulfilling requirements for treatment resistant depression (TRD). The present study is a secondary analysis of the STAR*D data conducted to compare the humanistic outcomes in patients with TRD and non-TRD MDD.

**Methods:**

Patients with major depressive disorder who entered level 3 of the STAR*D were included in the TRD group, while patients who responded to treatment and entered follow-up from level 1 or 2 were included in the non-TRD group. The first visit in level 1 was used for baseline assessments. The time-point of assessments for comparison was the first visit in level 3 for TRD patients (median day: 141), and the visit closest to 141 ± 60 days from baseline for non-TRD patients. Outcomes were assessed by the 12-item Short Form Health Survey (SF12), 16-item Quality of Life Enjoyment and Satisfaction Questionnaire (Q-LES-Q), Work and Social Adjustment Scale (WSAS), and Work Productivity and Activity Impairment scale (WPAI). Scores were compared in a linear model with adjustment for covariates including age, gender, and depression severity measured by the 17-item Hamilton Rating Scale for Depression (HDRS17) and Quick Inventory of Depressive Symptomatology (QIDS).

**Results:**

A total of 2467 (TRD: 377; non-TRD: 2090) patients were studied. TRD patients were slightly older (mean age 44 vs 42 years), had a higher proportion of men (49% vs 37%, *p* < .0001), and baseline depression severity (HDRS17: 24.4 vs 22.0, p < .0001) vs non-TRD patients. During follow-up, TRD patients had lower health-related quality of life (HRQOL) scores on mental (30 vs 45.7) and physical components (47.7 vs 48.9) of the SF12, and lower Q-LES-Q scores (43.6 vs 63.7), greater functional and work impairments and productivity loss vs non-TRD patients (all *p* < 0.05).

**Conclusion:**

Patients with TRD had worse HRQOL, work productivity, and social functioning than the non-TRD patients.

## Background

According to the World Health Organization, more than 300 million people worldwide suffer from depression. Depression is a leading cause of disability and a major contributor to global disease burden [1]. By 2020, major depressive disorder (MDD) is expected to be the second global leading cause of disability. MDD exhibits more debilitating effects on physical, social, and emotional functioning compared to any other chronic medical illness [2]. Although, several therapeutic options have proven efficacious in the treatment of MDD, [3] about 30% of patients with MDD fail to respond to antidepressant therapy, a condition referred to as treatment resistant depression (TRD) [4–7]. Factors such as fewer interpersonal or economic resources, minority status, lower function and quality of life, poor social and family support, and treatment non-compliance contribute to TRD [8–10].

The National Institute of Mental Health (NIMH)-sponsored Sequenced Treatment Alternatives to Relieve Depression (STAR*D) is the largest and most comprehensive clinical trial conducted in real-world settings of psychiatry and primary care to date, and included patients with nonpsychotic MDD [11]. In STAR*D study, patients were treated sequentially with a series of antidepressants or psychotherapy trials and the resistance was found to be increasing at Level 3 (failure of 2 therapies). Therefore, Conway et al. recently proposed an operational definition of TRD i.e., the failure of 2 adequate dose-duration antidepressants from different classes and/or psychotherapeutic treatments (either in combination or succession) in the current episode [5]. We have used a similar definition for TRD and used data from STAR*D study.

Humanistic outcomes as measured by health-related quality of life (HRQOL), functional and work productivity instruments, characterize the patient’s experience with the medical care. HRQOL equals perceived physical and mental health over time, and incorporates domains related to physical, mental and emotional, and social functioning. In addition to conventional clinical measures of health, HRQOL is increasingly used for assessing the quality of care in outcomes research [12].

It is well-known that depression has a debilitating effect on HRQOL [2, 13]. Symptoms of depression are associated with significant interference with functioning including absence from work, productivity loss, and lower job retention, resulting in an increased indirect cost [14, 15]. Patients with TRD have greater healthcare resource utilization and experience more difficulties in social and occupational function and a larger decline in physical health compared with other MDD patients [16]. The repeated and continuous symptoms of depression and associated distress experienced by TRD patients, and the associated social morbidity and chronic suffering, can infer vast socio-economic implications [17, 18].

Only a few studies have assessed the HRQOL in patients with TRD, [19–21] however, to our knowledge, none of the studies has compared the humanistic outcomes in TRD and non-TRD patients using a larger cohort from a real-world setting. Therefore, this study was conducted to evaluate various HRQOL and work productivity domains in patients with TRD versus those with non-TRD MDD using the STAR*D database.

## Methods

### Data source and study population

In the STAR*D study, outpatients from mental health and primary care practices, aged between 18 to 75 years, who met the Diagnostic & Statistical Manual Disorders (DSM-IV) criteria and had a 17-item Hamilton Rating Scale for Depression (HDRS17) [22] score ≥ 14 for nonpsychotic MDD were enrolled. Patients with bipolar or psychotic disorders, primary diagnosis of obsessive-compulsive disorder or an eating disorder, general medical conditions that contraindicated protocol medications in the first two treatment steps, substance dependence that required inpatient detoxification, and suicidal patients who required immediate hospitalization were excluded [23].

All patients provided written informed consent at study entry, at entry into each level, and the follow-up phase. For the present analysis, the study team obtained the limited access STAR*D dataset, following the NIMH procedure for obtaining and analyzing the research data [24]. As this was a retrospective analysis, so the institutional review board approval and informed consent were not required. Patient identifiers are not disclosed and only summary data are presented.

### STAR*D study design

A detailed description of STAR*D study design has been presented elsewhere [23]. The STAR*D was a prospective, pragmatic clinical trial conducted at multiple sites in the United States that evaluated the relative efficacy and tolerability of various antidepressants in outpatients with nonpsychotic MDD [25, 26]. All patients started with a single selective serotonin reuptake inhibitor (SSRI) (citalopram) and followed an algorithm-based acute phase treatment over a 12-week period. Patients who did not have remission after the initial treatment, participated in a sequence of up to three randomized trials (Levels). Patients who achieved remission or a response with an adequate benefit according to clinician’s judgment after any of the treatment levels could enter the 12-month naturalistic follow-up phase. Patients were allowed to choose among acceptable treatment options reflecting the clinical practice. Patients could switch to bupropion, venlafaxine, sertraline, cognitive therapy (Level 2), mirtazapine, nortriptyline (Level 3), tranylcypromine, mirtazapine+venlafaxine (Level 4) or augment the current treatment with bupropion, buspirone, cognitive therapy (Level 2), lithium, T3 thyroid hormone (Level 3). There were no meaningful clinical differences due to pharmacological differences between treatment options and probability of remission was not clearly dependent on choice of medication [25] (Table 1).

**Table 1:** STAR*D study design and categorization of patients (TRD vs non-TRD)

	Non-TRD			TRD	
	Non-TRD 1	Non-TRD 2		TRD1	TRD2
	Patients Entered Follow-up after	Patients Entered Follow-up after	Patients Entered Follow-up after	Patients Entered	Patients Entered
	Level 1	Level 2	Level 2A	Level 3	Level 4
Level 1 Baseline	X	X	X	X	X
Level 2		X	X	X	X
Level 2A			X	X	X
Level 3				X	X
Level 4					X
Follow-up	X	X	X	X	X

*TRD* treatment resistant depression

A detailed description of STAR*D study design and different levels of treatment has been presented elsewhere: Rush AJ, Fava M, Wisniewski SR et al. Sequenced treatment alternatives to relieve depression (STAR*D): rationale and design. Control Clin Trials 2004; 25: 119–42

The present study is a secondary analysis based on data collected in the STAR*D. For the present study, TRD and non-TRD MDD patients were compared. Patients who entered level 3 of the STAR*D trial (i.e. failed to remit or achieve adequate response after two antidepressant trials) were included in the TRD group, while patients who entered follow-up after level 1 or level 2 (or 2A) and were included in the non-TRD group. The first visit in level 1 was used for baseline assessments. Comparison of outcome measures between TRD and non-TRD groups was made at primary visits which for TRD patients was the first visit in level 3. The median day of the primary visit for TRD patients was 141, therefore, the visit closest to 141 days from baseline with a deviation ±60 days was considered the primary visit for a non-TRD patient. Treatment response of patients in both the cohorts was also observed at a longer duration including at 12-month, considering a window period of 365 ± 60 days. However, due to low number of patients in both the cohorts (TRD: 28; non-TRD: 16), no analysis was performed.

### Assessments

#### Demographics and baseline clinical characteristic

Demographics and baseline clinical characteristics were assessed at the first visit of level 1 using HDRS17, [22] the 14-item Cumulative Illness Rating Scale (CIRS), [27] and the 16-item Quick Inventory of Depressive Symptomatology (QIDS) Self-Reported (QIDS-SR16) and Clinician-rated (QIDS-C) versions [28]. To equate HDRS17 total scores indicating no depression (score = 0–7), mild depression (score = 8–13), moderate depression (score = 14–19), severe depression (score = 20–25), and very severe depression (score = 26+) with QIDS-SR16 total scores, a conversion table was used to provide equivalent QIDS-SR16 ratings (no depression: score = 0–5; mild: score = 6–10; moderate: score = 11–15; severe: score = 16–20; very severe: score = 21+).

#### Outcome assessments at primary visit

The HRQOL was measured using the Short Form Health Survey (SF-12) and the Quality of Life Enjoyment and Satisfaction Questionnaire (Q-LES-Q). The SF-12 is a 12 item, self-report instrument that assesses mental and physical health status [29], while the 16-item short version of Q-LES-Q, a self-report instrument, was used that measures the degree of enjoyment and satisfaction experienced by patients in several domains of functioning (e.g., physical health, feelings, work, household duties, school/house work) [30].

Functioning was measured using the Work and Social Adjustment Scale (WSAS), and the Work Productivity and Activity Impairment scale (WPAI). The WSAS is a 5 item self-reported instrument that measures functional impairment (the ability to work, manage home, social and personal leisure activities, and the ability to form and maintain close relationships) and the WPAI is a six-item self-report questionnaire that measures the number of work hours missed or the number of hours worked in the past 7 days, and impairment resulting from health conditions while working or performing usual daily activities other than work.

### Statistical analysis

The sample size of this study was not calculated based on any statistical consideration, however all patients with measurements available at both baseline and primary visits were included in the analysis. Demographics and baseline clinical characteristics were summarized descriptively in each group using mean and standard deviations (SD) for continuous variables and frequencies for categorical variables. Association of baseline clinical characteristics with TRD was investigated by t-test or logistic regression models. Humanistic outcomes were compared between TRD and non-TRD patients using a linear model adjusting for the covariates that could potentially affect the outcome such as baseline of the variable, age category, gender, and baseline values of total severity score of comorbidity, Depression severity by HDRS17, and Depression severity by QIDS. Missing values were not imputed, as the exact reason for missing data in the STAR*D study was not clear.

## Results

### Demographics and baseline clinical characteristic

Out of 3671 patients who entered level 1, 2467 (67%) patients with both baseline and first visit assessments at level 3 (or around 141 days) were included in the analysis. The remaining 1204 patients were lost to follow up. Of the 2467 patients included in the analysis, 377 entered level 3 (TRD group), while 2090 entered follow-up from levels 1 and 2 (non-TRD group). (Table 1).

The TRD patients were slightly older than the non-TRD patients (mean [SD] age 44 [11.97] vs 42 [13.26] years, *p* = .0005). The TRD group had a higher proportion of men compared with the non-TRD group (49% vs 37%, *p* < .0001). Patients with TRD had higher scores of HDRS17 (24.4 vs 22.0, p < .0001) and QIDS-SR16 (17.0 vs 14.7, p < .0001) compared with the non-TRD patients. More patients in the TRD group than in non-TRD group had a very severe depression as measured by HDRS17 (40% vs 22%) and QIDS-SR16 (20% vs 10%). In general, TRD patients were observed with either comparable or worse depression scores compared to non-TRD patients at baseline (Table 2).

**Table 2:** Demographics and baseline clinical characteristics of TRD and non-TRD patients

Variable	N	TRD	N	Non-TRD	*P*-Value
Age at entry to study; mean years (SD)	377	44.3 (11.97)	2090	41.8 (13.26)	0.0005
Age, years; (%)					0.0004
18–34	98	26	734	35.1	
35–49	145	38.5	744	35.6	
50–64	122	32.4	512	24.5	
65+	12	3.2	100	4.8	
Gender; n (%)					<.0001
Female	193	51.2	1322	63.2	
Male	184	48.8	769	36.8	
HDRS17 current score (transcribed); mean (SD)	377	24.4 (5.10)	2089	22.0 (4.80)	<.0001
Depression severity by HDRS17; (%)					<.0001
Mild (score: 8–13)	0	0	3	0.1	
Moderate (score:14–19)	63	16.7	703	33.7	
Severe (score: 20–25)	164	43.5	920	44	
Very severe (score:26+)	150	39.8	463	22.2	
QIDS-C current score (transcribed); mean (SD)	377	17.5 (3.19)	2089	15.8 (3.27)	<.0001
Depression severity by QIDS-SR16; (%)					<.0001
No Depression (score:0–5)	0	0	25	1.2	
Mild (score:6–10)	18	4.8	308	14.8	
Moderate (score:11–15)	115	30.6	861	41.3	
Severe (score:16–20)	168	44.7	693	33.3	
Very severe (score:21+)	75	19.9	197	9.5	
QIDS-SR current score (transcribed); mean (SD)	376	17 (3.86)	2084	14.7 (4.25)	<.0001
Total severity score of comorbid condition; mean (SD)	377	5.6 (4.43)	2091	4.3 (3.66)	<.0001

*HDRS17* The Hamilton Rating Scale for Depression, *QIDS-C* Quick inventory of depressive symptomatology (clinician-rated), *QIDS-SR* Quick inventory of depressive symptomatology (self-rated), *SD* Standard deviation, *TRD* Treatment resistant depression

### Humanistic outcomes

#### HRQOL

The number of patients observed at the primary visit for all the outcome measures varied from those at baseline, as not all data were collected at every visit for all patients. Majority of the patients (*n* = 316) with both baseline and primary visit values were observed for outcome based on SF12 measurement. The median and mean (standard deviation) day of primary visit was 133 and 136 (37.9) for non-TRD patients, respectively. Patients with TRD had significantly lower scores on the mental component (*p* < .0001) and physical component (*p* = 0.0126) of the SF-12 scale compared with non-TRD patients being at the same time window. The TRD patients also reported lower Q-LES-Q global scores compared with non-TRD patients (*p* < .0001) (Table 3).

**Table 3:** Quality of life and functional impairment at Primary Visit^a^ for TRD and non-TRD patients

Endpoint	TRD	Non-TRD	Mean Difference TRD vs non-TRD	95% CI	*P*-value
Quality of Life, mean					
Short form (SF-12) scores^c^					
Mental component	30.0	45.7	−15.7	−17, −14.4	<.0001
Physical component	47.7	48.9	−1.2	−2.1, −0.3	0.0126
Q-LES-Q General activities index	43.6	63.7	−20.1	−22.2, −18.1	<.0001
Functional impairment					
WSAS score	23.4	11.3	12.0	10.9, 13.2	<.0001
WPAI, %^b^					
Activity impairment	54.5	30.5	24.0	20.8, 27.2	<.0001
Percent hours missed	19.6	9.5	10.1	6.4, 13.8	<.0001
Work impairment	43.0	19.2	23.8	19.7, 27.9	<.0001
Overall work impairment	52.9	24.9	28.0	23, 33	<.0001

^a^ The primary visit is the first visit in level 3 for TRD patients and the visit closest to 141 days from baseline visit and with a deviation ≤60 days for non-TRD patients

Least square means, 95% CIs, and *p*-values were obtained from a linear model adjusted for baseline of the variable, age category, gender, and baseline values of total severity score of comorbidity, Depression severity by HDRS17, and Depression severity by QIDS-SR16

^b^WPAI scores are based on 7-day recall period

*SFHS* Short form health survey, *Q-LES-Q* Quality of life enjoyment and satisfaction, *WSAS* Work and social adjustment scale, *WPAI* Work productivity and activity impairment, *TRD* Treatment resistant depression, *CI* Confidence interval

^c^Range of SF-12 scores reported: Mental Component 13.8 to 67.9; Physical Component 7.4 to 70.5. Lower score indicates poorer mental or physical health-related function and wellbeing

#### Work and social functional impairment

At the time of meeting the TRD criteria, patients in the TRD group reported greater functional impairments in work and social functioning compared with the non-TRD group. TRD patients had higher scores at WSAS and all scales of WPAI compared with the non-TRD group (*p* < .0001) (Table 3), indicating greater functional and work impairments, and higher productivity loss due to health.

## Discussion

This study shows that patients meeting the TRD criteria in the STAR*D had worse HRQOL scores, work productivity, and greater functional impairments compared to non-TRD patients. At baseline, the TRD patients exhibited greater depression severity, however the quality of life and functional parameters were equal in both the cohorts. The difference in humanistic outcomes several months later suggests a decrease in quality of life and functioning, over time in patients with depression that is not alleviated in comparison to those effectively treated. To our knowledge, the present study is the first to compare the humanistic outcomes in TRD with non-TRD patients using a large dataset and a working definition of TRD [5].

In the present study, patients with TRD had poorer HRQOL scores compared with non-TRD patients, as measured by the SF-12. A few studies have evaluated the screening performance of the mental health component of SF-12 and suggested a cutoff value of 42 [31] or 45.6 as the best screening cutoff for depression [32]. In the present study, patients with TRD had lesser mental health component scores (30) than these cutoffs. However, the scores in non-TRD patients (45.7) were almost equal to at least one of the suggested cutoffs. The physical health component scores in both TRD and non-TRD groups were comparable suggesting a greater impairment in mental health of TRD patients compared to physical health impairment.

A previously published study used STAR*D data to assess the HRQOL of patients with MDD using Q-LES-Q. In that study, it was found that patients who did not remit or achieve adequate response to first line selective serotonin reuptake inhibitor treatment had poor Q-LES-Q scores which, while improved after second line therapy, however still failed to achieve normal scores [33]. The Q-LES-Q scores observed in the present study also indicate a generally poor HRQOL status in both TRD and non-TRD patients. However, the scores were significantly worse in TRD patients compared with non-TRD patients.

In the present study, it was found that patients with TRD had significantly greater functional impairments compared with non-TRD patients as measured by WSAS and WPAI scores. This finding is in agreement with a Canadian study [21] of outpatients with various depressive conditions, which found that patients with TRD had greater functional impairments when compared to patients with treatment responsive depression. Another study [20] showed that patients with primary unipolar major depression who achieved remission with residual symptoms had a longer period of impairment in occupational functioning, with worse overall scores on the Social Adaptation Scale and the Global Assessment of Functioning, compared to those who had remission without residual symptoms.

Generally, in the assessment of mental disorders, more importance has been given to management of symptoms rather than functional impairment [34]. The traditional HRQOL scales were based on symptomatic assessments made by a single respondent (either patient or physician). However, an emerging consensus has been developed in considering the patient’s perspective related to functional impairment as an important aspect in monitoring and evaluating HRQOL outcomes [34, 35]. Thus, an increasing importance in the assessment of patient’s perspective on impairments in addition to symptoms is needed.

We used patient-reported outcome (PRO) data from the STAR*D study to compare various aspects of humanistic burden in TRD and non-TRD patients. The STAR*D study was the first major study that investigated the effectiveness of treatments in outpatients with nonpsychotic MDD who did not achieve an adequate response after an initial antidepressant trial. The STAR*D study was designed to achieve more generalizability by including a more representative population, using minimal exclusion criteria and keeping the treatments unblinded [23]. Therefore, the results of the present study may be generalizable to the overall humanistic burden in TRD and non-TRD patients.

The use of STAR*D data may have limitations. As the STAR*D study was completed in 2006, the results do not fully reflect current medical practice and healthcare policies. It has been reported that TRD patients in the STAR*D study had higher rates of psychiatric comorbidities, [10] and the status of comorbidities or the association of comorbidities with clinical severity, HRQOL, and functional impairment was not addressed in our study. Also, since this is a secondary analysis of the STAR*D and based on a subgroup of patients (patients who entered level 3 of the STAR*D), there may be some selection bias.

Severity of illness, age at onset of MDD, ethnicity, marital status, employment status, educational level, and a number of other sociodemographic factors have been found to be associated with several domains of HRQOL in patients with depression [17, 36]. For instance, increased comorbidities, fewer years of education, unemployment, or belonging to a minority group were associated with worse physical and mental functions on the HRQOL domains [17, 36]. Since the baseline characteristics in our study were not balanced due to lack of randomization, it could have been a source of potential confounding. However, we adjusted the estimates for demographic and clinical characteristics including age, gender, the CIRS, HDRS17 and the QIDS-SR16. Additionally, as we did not assess any causal association, we can consider both the possibilities that it is the humanistic outcomes that interfered with the treatment effect or lack of effective treatment worsened humanistic outcomes.

## Conclusion

The findings of the present study expand the evidence that patients with TRD experience greater humanistic burden measured as HRQOL, work and social functioning and work productivity compared with non-TRD patients. This highlights the humanistic burden of TRD, and its potential impact on the individual patient as well as on societal burden and costs. Further measures should be taken to limit the humanistic as well as the clinical and economic consequences of TRD.

## Abbreviations

HRQOL: Health-related Quality of Life
MCS: Mental Component Summary
MDD: Major depressive disorder
NIMH: National Institute of Mental Health
PCS: Physical Component Summary
PRO: Patient-reported outcome
QIDS: Quick Inventory of Depressive Symptomatology
TRD: Treatment Resistant Depression
WPAI: Work Productivity and Activity Impairment
WSAS: Work and Social Adjustment Scale
